# Type 2 Decompensated Diabetes Mellitus Weaned off Subcutaneous Insulin Therapy by Utilizing a Continuous Glucose Monitoring Device

**DOI:** 10.7759/cureus.36970

**Published:** 2023-03-31

**Authors:** AndreyI Manov, Gundip S Dhillon, Sukhjinder Chauhan, Blerina Asllanaj, Erie S Uy

**Affiliations:** 1 Internal Medicine, MountainView Hospital, Las Vegas, USA

**Keywords:** type 2 diabetes, serum blood glucose, insulin injection, oral anti-diabetic agents, continuous glucose monitoring systems

## Abstract

A case series was conducted on three patients diagnosed with decompensated type 2 diabetes mellitus (T2DM) who had hemoglobin A1c (HbA1c) levels ranging from 9.5% to above 14%. Patients were self-monitoring blood glucose (SMBG) levels four times a day. These patients were seen at the resident continuity clinic and were placed on continuous glucose monitor (CGM) devices to monitor their blood glucose levels. To improve the effectiveness of the treatment closely, a CGM team consisting of transitional year and internal medicine residents was arranged.

The CGM team provided comprehensive education and written instructions on dietary changes, insulin administration, and physical activity at monthly follow-up appointments. Before the instructions were given to the patients, they were reviewed and approved by the supervising attending physician who was a board-certified endocrinologist. Our CGM team successfully managed these three patients with T2DM by tailoring their insulin regimens by using real-time CGM data. With the help of close CGM monitoring, patients were successfully transitioned from requiring multiple subcutaneous insulin injections to oral anti-diabetics. After the transition, patients’ T2DM remained well-controlled with an HbA1c level of less than 7% at their follow-up appointments.

This case series demonstrated the successful implementation of CGM-guided T2DM treatment in a continuity clinic managed by residents. To our knowledge, the use of CGM-guided T2DM treatment in the setting of resident care has never been reported in the United States before. This may serve as a benchmark for other continuity clinics which residents run across the country.

## Introduction

Approximately 90 million people in the United States have pre-diabetes, while 34 million have been diagnosed with diabetes mellitus. The majority of those diagnosed (90-95%) have type 2 diabetes mellitus (T2DM), while 5-10% have type 1 diabetes mellitus (T1DM) or other rare forms of the disease. The condition is a major cause of blindness, end-stage renal disease (ESRD), and non-traumatic amputations, and greatly contributes to deaths due to macrovascular disease [1,2].

Managing diabetes mellitus is difficult and requires patients to make dietary changes, monitor glucose levels, and engage in regular physical activity. Patients with T2DM and HbA1c levels greater than 9% may need initial treatment with insulin regimens, typically combined with oral diabetic medications such as GLP-1 receptor agonist injections (GLP-1-RAG), especially when associated with cardiovascular diseases [1,2]. Patients who require insulin for their initial treatment are at high risk for hypoglycemic or hyperglycemic events. To prevent such adverse events secondary to insulin use, they must self-monitor their blood glucose four times a day, which can be burdensome and leads to poor adherence. The self-monitoring blood glucose (SMBG) four times daily only provides a snapshot of the serum glucose level at one point in time. The SMBG makes proactive management of blood glucose levels through self-monitoring a difficult task [3-5].

In this case series, we present three patients with decompensated T2DM on insulin therapy with hemoglobin A1c (HbA1c) levels ranging from 9.5% to 14% and self-monitoring their blood glucose levels. These patients were initiated on a continuous glucose monitor (CGM) device. We have decided to introduce CGM technology to general internal medicine continuity clinics, which are managed by residents under the supervision of an endocrinologist. It's worth noting that this move is significant since it is the first time that CGM technology is being implemented in continuity internal medicine clinics managed by residents under the supervision of endocrinologists, whereas typically CGM devices are monitored in specialized endocrinology clinics [6].

CGM employs a device that measures glucose levels in interstitial fluid and transmits real-time data wirelessly. To ensure optimal glycemic control, the American Diabetes Association (ADA) suggests monitoring hemoglobin A1c, time in range (TIR), and glucose management indicator (GMI). GMI is a calculated metric based on the weighted average of glucose readings over time, providing a detailed view of glycemic control. Monitoring GMI via CGM facilitates informed decision-making for better management of diabetes [7].

## Case presentation

Case 1

A 71-year-old African American female presented at the clinic at the end of 2020 with decompensated T2DM and multiple other medical problems, including heart failure with preserved ejection fraction (HFPEF), secondary adrenal insufficiency, osteoporosis, major depressive disorder, hypertension, and painful diabetic neuropathy. Her blood sugars had been ranging between 250 and 400 mg/dL and her HbA1c was 11%. Per Table 1, patients' GMI was tracked while they were on CGM, and medications were adjusted accordingly.

**Table 1:** Case 1 - GMI readings after months of using CGM. CGM: continuous glucose monitoring; GMI: glucose management index

Months	GMI (glucose measurement index)
Month 0	11% (5.7-6.3%)
Month 3	6.5 (5.7-6.3%)
Month 11	6.2% (5.7-6.3%)
Month 18	6.6% (5.7-6.3%)

At the initial visit, her basal-bolus insulin regimen was adjusted to long-acting insulin glargine 20 units twice a day (BID) and rapid-acting insulin aspart 10 units before meals, and her pioglitazone was stopped due to recently decompensated HFPEF and replaced with canagliflozin 100 mg once a day. The patient was placed on a CGM device at that visit. She was educated on how to titrate her insulin by monitoring her blood glucose with the help CGM device and educated on maintaining a healthy lifestyle with dietary recommendations. After a month of follow-up, her blood glucose levels started to improve and her fasting serum blood glucose was mostly ranging from 93 to 100 mg/dL with some lows in the 50s to 60s mg/dL. Based on this data, her basal insulin glargine was down-titrated from 20 units to 12 units BID and her rapid-acting aspart insulin was decreased to 4 units before each meal.

The patient was seen at the clinic every month. The CGM team contacted her twice a week to adjust her insulin regimens based on the CGM data. After three months of using the CGM, her GMI was 6.5% and her insulin and canagliflozin were discontinued. The patient was started on dulaglutide 0.75 mg subcutaneously once a week, and 11 months later, her GMI was 6.1% with TIR 73%.

In the spring of 2022, the patient was started on empagliflozin 10 mg once a day and oral semaglutide tablets 3 mg once a day, which was slowly titrated to 7 mg once a day on subsequent follow-up visits. The patient was followed in the clinic every month for 18 months, and her GMI remained below 6.6%.

Case 2

A 67-year-old male with a history of hypertension and T2DM was initially admitted to the hospital in the autumn of 2021 due to increased fatigue, polyuria, and decreased appetite over the last two days. He was diagnosed with moderately severe diabetic ketoacidosis (DKA). The patient had been treated for type 2 diabetes mellitus for three years with metformin 1 g BID and an American Diabetes Association (ADA) diet of 1800 calories, but he had not been compliant with his treatment plan and had not been following up on his blood sugar levels.

During hospital admission, laboratory studies revealed a glucose level of 297 mg/dL, pH of 7.20, beta-hydroxybutyrate of 7 mmol/L, albumin-adjusted anion gap of 18, and serum bicarbonate of 11 mmol/L. The patient was treated for DKA with intravenous fluids, potassium, and magnesium replacements, and IV insulin. He was discharged with a regimen of basal insulin glargine 16 U at night, rapid-acting insulin lispro before meals, and correctional doses of rapid-acting insulin lispro based on his pre-prandial blood sugar. At the time of discharge, he was injecting 35 units of basal-bolus and correctional insulin for 24 hours to control his blood sugar. Per Table 2, patients' GMI was tracked while they were on CGM, and medications were adjusted accordingly. 

**Table 2:** Case 2 - GMI readings after months of using CGM. CGM: continuous glucose monitoring; GMI: glucose management index

Months	GMI (glucose measurement index)
Month 0	7% (5.7-6.3%)
Month 3	6.5% (5.7-6.3%)
Month 6	5.6% (5.7-6.3%)
Month 9	6.6% (5.7-6.3%)

About a month after discharge from the hospital patient patiently followed up at the continuity clinic. His insulin regimen was adjusted to insulin glargine 16 units before bed and 6 units of insulin lispro before meals. The importance of diet and exercise was emphasized, and the patient was started on a CGM device. Based on the shared CGM data, insulin doses gradually decreased.

In early 2022, the patient's GMI was 7% with minimal insulin doses. The insulin therapy was discontinued, and the patient was transitioned to empagliflozin 10 mg/day, semaglutide 0.25 mg subcutaneous injection once a week, and glimepiride 2 mg/day. At the monthly follow-up after transition, the patient's GMI dropped to 6.5% with an average blood glucose level of 134 mg/dL. The TIR was 90% and the coefficient variable (CV) was 26%, with only 10% of readings being high and none considered very high. No instances of low or very low blood sugar were recorded. At subsequent follow-up visits, semaglutide subcutaneous injection was discontinued and oral semaglutide tablets at a dosage of 3 mg/day were started. The patient continued to take glimepiride (2 mg/day) and semaglutide was up-titrated to 7 mg/day as part of his regimen. After another three months of follow-up, the patient’s GMI decreased from 6.5% to 5.6%, and the glimepiride was discontinued.

By the autumn of 2022, the patient's average blood glucose over a 90-day period was 136 mg/dL, CV was 28%, TIR was 94%, and there were no cases of very high or low/very low blood glucose. However, her GMI at this time increased from 5.6% to 6.6% after the discontinuation of glimepiride. The patient was thus started with empagliflozin 10 mg. The patient was monitored at follow-up visits, and her HbA1c levels stayed below 7% and she continued to take her oral diabetic medications.

Case 3 

A 42-year-old Hispanic male presented to our continuity clinic to establish care at the beginning of 2022. His past medical history was remarkable for decompensated T2DM with an HbA1c above 14%. His self-monitored blood glucose readings at home were between 250 and 500 mg/dL. He was previously treated with metformin ER 750 mg/day by another physician. Other medical history was remarkable for class 2 obesity with a BMI of 35 kg/m^2^, hypertension controlled with lisinopril, obstructive sleep apnea treated with CPAP, and hyperlipidemia treated with atorvastatin daily.

On that visit, he was started on insulin glargine before bedtime, rapid-acting insulin lispro before meals, and metformin ER 2000 mg/day. The risks and benefits of CGM device placement were discussed with the patient. The patient agreed with the placement of the CGM device and shared his data with the clinic. The CGM team educated him on lifestyle medications including dietary changes, actively participating in daily physical activity, and how to adjust insulin doses based on his blood glucose levels monitored by the CGM device. The patient was also started on extended-release topiramate and phentermine hydrochloride for class 2 obesity. The CGM team contacted him twice a week to adjust his insulin regimens based on the CGM data and he was scheduled to follow up monthly in the clinic. Per Table 3, patients' GMI was tracked while they were on CGM, and medications were adjusted accordingly.

**Table 3:** Case 3 - GMI readings after months of using CGM. CGM: continuous glucose monitoring; GMI: glucose management index

Months	GMI (glucose measurement index)
Month 0	14% (5.7-6.3%)
Month 1	8.2% (5.7-6.3%)
Month 3	7.4% (5.7-6.3%)
Month 9	6.9% (5.7-6.3%)

A month after his initial visit, the patient's GMI was recorded to be 8.2% which down trended from HbA1c of 14%, and his average blood glucose level was 185 mg/dL. At this visit, rapid-acting insulin was discontinued, the patient remained on a basal regimen of insulin glargine at bedtime and metformin 2000 mg/day and was started on semaglutide 0.25 mg subcutaneous injection once a week.

Three months later, the CGM data were reviewed at the clinic and the patient's GMI improved from 8.2% to 7.4%. At this visit, the basal insulin regimen was discontinued, the patient remained on metformin 2000 mg, semaglutide was up-titrated from 0.25 mg to 1 mg subcutaneous injections once per week, and empagliflozin 10 mg was added to this regimen. On subsequent follow-up visits, the patient's GMI remained below 6.9% with an average blood glucose of 151 mg/dL, TIR of 85%, and no instances of very high or low blood glucose were recorded. The patient's T2DM was well controlled with oral anti-diabetic medications and semaglutide injection once a week.

## Discussion

Over the past decade, advancements in technology have greatly impacted diabetes management with the use of CGM devices. CGM devices have become more compact, and accessible through a wider range of insurance plans.

Comparative studies between continuous glucose monitoring using CGM devices and self-monitoring of blood glucose have been conducted in patients with T1DM which have shown a significant decrease in patients’ HbA1c levels who were using CGM devices [7-9]. Both randomized and observational studies have shown that real-time CGM systems lead to better glucose control as measured by TIR, reduced glucose variability as measured by the CV, and a reduction in both hyperglycemia and hypoglycemia for individuals with type 1 and insulin-requiring type 2 diabetes who receive multiple insulin injections daily [7-11].

The GMI, a measure derived from average glucose readings, provides an estimation of HbA1c levels that is less prone to interference from conditions, such as anemia, chronic kidney disease, polycythemia, and liver cirrhosis [11]. The American Diabetes Association (ADA) recommends blood glucose control using CGM [12,13].

The aim is to maintain a TIR between 70 and 180 mg/dL, which should account for 70% of the time, except in the case of older or pregnant patients. Studies have demonstrated a correlation between TIR and the development of microvascular complications, such as retinopathy and diabetic nephropathy, as indicated by microalbuminuria in patients with diabetes mellitus [14].

CGM devices have proven to be valuable tools for 24-hour blood glucose monitoring which has led to improvements in average HbA1c levels by 0.3-0.6% and a decrease in the incidence of hypoglycemia in patients who were on insulin [15,16]. The CGM devices have also significantly improved patient satisfaction compared to traditional SMBG using fingersticks daily. With real-time glucose monitoring via the utilization of CGM devices, patients improved their eating patterns, physical activity, and overall quality of life [17-21].

Based on this case series of three patients, our results suggest that prompt treatment with insulin while monitoring their blood glucose levels using CGM devices can effectively combat glucotoxicity and allow T2DM patients to attain optimal glucose control. Sharing their CGM data with our continuity clinic, allowed us to make a more accurate adjustment to their insulin regimen. Although our study was limited to only three patients, this may suggest that further studies need to be made using CGM. Insulin therapy in patients with T2DM may be a temporary measure to improve glucotoxicity and allow the pancreas' beta cells to respond to oral anti-diabetic drugs and or injectable GLP-1 receptor agonist injections (GLP-1-RAG).

## Conclusions

The results of this case series highlight the importance of CGM in treating patients with type 2 diabetes mellitus. With the ability to see real-time glucose levels and trends, patients can make more informed decisions about their diet, exercise, and medication regimens. Based on this study, CGM use needs to be further studied in order to better manage patients' glucose and improve their health outcomes. Moreover, the use of CGM can also provide valuable insights for healthcare providers. The data collected through CGM can be used to fine-tune treatment plans and adjust as needed, helping to ensure that patients are receiving the best possible care.

The successful implementation of CGM at a continuity clinic managed by residents and supervised by a board-certified endocrinologist shows the potential for widespread adoption of this approach. This case series serves as a model for other internal and family residency programs to follow, offering improved care for patients with T2DM but also educational benefits for future healthcare providers. By incorporating CGM-guided T2DM, future physicians can play a crucial role in better managing and ultimately defeating this debilitating disease. To further validate this approach, similar pilot programs should be funded by the US government. Once further studied this concept can be tested and implemented on a larger scale at continuity clinics managed by residents under supervision.
